# Integrating network pharmacology and transcriptomics to identify solasonine’s anti-osteosarcoma targets and experimental validation

**DOI:** 10.3389/fonc.2025.1614058

**Published:** 2025-08-04

**Authors:** Daihao Wei, Minyu Li, Dawei Chu, Jiandang Shi

**Affiliations:** ^1^ Department of Orthopedic, General Hospital of Ningxia Medical University, Yinchuan, Ningxia Hui Autonomous Region, China; ^2^ The First School of Clinical Medicine, Ningxia Medical University, Yinchuan, Ningxia Hui Autonomous Region, China

**Keywords:** solasonine, osteosarcoma, network pharmacology, transcriptomics, experimental validation

## Abstract

**Background:**

Osteosarcoma (OS) patients face the challenge of having few effective therapeutic drugs. Solasonine(SS)is an active component of TCM against OS cells. This study aims to identify the key targets of solasonine in treating OS.

**Methods:**

In this study, the transcriptome data and related gene sets were first downloaded from public databases. Subsequently, candidate targets were obtained by intersecting differentially expressed genes (DEGs) with solasonine and OS disease targets. Key targets were then identified through regression analyses, and a prognostic model was constructed. A nomogram was subsequently constructed using the key targets. The functions and immune microenvironment, as well as the structure, regulatory network, and molecular docking of these key targets, were then analyzed. The expression level of the candidate targets in osteosarcoma cells was verified in RT-qPCR experiments, and the effect of solasonine on the malignant biological behavior of osteosarcoma cells was verified.

**Results:**

DEGs, targets corresponding to solasonine, and OS-related disease targets were intersected to obtain 37 candidate targets. Subsequent regression analyses identified 5 key targets (ATP1A1, CLK1, SIGMAR1, PYGM, HSP90B1). It was further demonstrated that the OS prognostic model constructed using these key targets was robust. The constructed nomogram provided an excellent predictive model. Moreover, some pathways, such as cytokine-cytokine receptor interaction, were significantly enriched, and there were 4 significantly different immune cells and 3 significantly different immune checkpoints (*P<0.05*). Additionally, natural killer cells and activated B cells were significantly positively correlated (cor = 0.68, *P < 0.001*). The subsequent regulatory network included transcription factors regulating the 5 targets. All key targets showed favorable molecular docking effects with SS. The target genes all exhibited higher expression in osteosarcoma cell lines(*P<0.05*). Solasonine can inhibit the malignant biological behavior of cell proliferation, migration and invasion.

**Conclusion:**

In this study, ATP1A1, CLK1, SIGMAR1, PYGM, and HSP90B1 were identified as key targets of solasonine in the treatment of OS, and they were found to have reference significance for the treatment of OS. SS can be a potential drug for the treatment of osteosarcoma.

## 1 Introduction

Osteosarcoma (OS) is a primary malignant bone tumor predominantly occurring in adolescents, characterized by a high tendency for recurrence and metastasis (1–5). Despite significant advancements in OS treatment globally, the 5-year survival rate for metastatic cases remains below 30% (6, 7). At present, the treatment of osteosarcoma involves surgery combined with chemotherapy. However, the high recurrence and metastasis rates of osteosarcoma limit the effectiveness of surgical intervention (8). Additionally, chemotherapy-related side effects and drug resistance persist as significant clinical challenges. OS continues to face issues such as high metastasis rates, limited effective therapeutic drugs, a unique tumor microenvironment, high heterogeneity, and a lack of specific therapeutic targets (9). These challenges underscore the need to identify potential drug-active components as novel therapeutic agents for OS. Traditional Chinese Medicine (TCM) has been used in China for thousands of years, and its antitumor effects, as well as its ability to enhance efficacy and reduce toxicity, have been increasingly validated (10). Solanum nigrum L. (Long Kui), a traditional Chinese medicine, is known for its anti-inflammatory, swelling-reducing, and anti-tumor properties (11). Solasonine (SS), a steroidal glycoalkaloid, is one of the primary active components of the traditional Chinese medicine Long Kui. It has been shown to have anti-tumor effects in various cancers, including prostate cancer, liver cancer, breast cancer, and bladder cancer. The chemical structure of solasonine is C45H73NO16, consisting of a steroidal alkaloid aglycone and a sugar chain. Solasonine has various pharmacological effects such as anti-tumor, anti-inflammatory and neuroprotection (12). In several studies, solasonine can induce apoptosis of cancer cells and inhibit the proliferation of tumor cells (13, 14). Solasonine can regulate the expression of Bax, Bcl-2, Bcl-xL and other proteins, change the mitochondrial membrane permeability, release cytochrome c, activate Caspase-3 and so on, induce a variety of tumor cells apoptosis,such as breast cancer Bcap-37 cells, lung cancer H446 cells (12, 15). Solasonine can induce ferroptosis in tumor cells. As an inducer of ferroptosis, solasonine can promote ferroptosis of hepatoma carcinoma cells via glutathione peroxidase 4-induced destruction of the glutathione redoxsystem (16). Although numerous studies have explored the anti-tumor effects of solasonine, very few have focused on its target in osteosarcoma, with only one relevant report. Wang et al. suggest that SS suppressed cancer stem-like properties and epithelial–mesenchymal transition (EMT) by inhibiting aerobic glycolysis in OS cells in an ALDOA-dependent manner (17). The literature does not provide detailed elaboration or research on the drug target. Therefore, considering the difficulty of treating osteosarcoma and the role of SS in tumor treatment, we need to continue exploring its pharmacological mechanisms, clarify its interactions with various biomolecules *in vivo*, identify the SS anti-osteosarcoma target and related molecular mechanisms, evaluate its safety, efficacy, and optimal treatment regimen, thus laying a solid foundation for the clinical application of solasonine in osteosarcoma.

Network pharmacology is centered on constructing networks that link drugs, targets, and diseases to systematically reveal the mechanisms of drug action and their multi-target characteristics (18, 19). The development of transcriptomics has empowered researchers to observe gene functions and regulatory networks from databases, uncovering intricate molecular interactions and signaling pathways (20). By integrating phenotypic and molecular-level information, transcriptomics offers new perspectives for fundamental biological research and disease mechanism studies. The integration of network pharmacology and transcriptomics enhances the systematic, accurate, and efficient screening of drug targets corresponding to diseases. This combined approach not only identifies potential therapeutic targets but also elucidates the underlying molecular mechanisms, paving the way for the advancement of more effective and targeted therapies.

Therefore, this study, based on network pharmacology combined with transcriptomic data, identified five key targets of solasonine against OS through differential gene screening and univariate, multivariate, and stepwise regression analyses. The five targets were ATP1A1, CLK1, SIGMAR1, PYGM, and HSP90B1. A prognostic model constructed using these key targets demonstrated their prognostic value, and subsequent analyses highlighted the significance of these targets. Furthermore, RT-qPCR experiments validated the expression levels of these targets in osteosarcoma cells. *In vitro* experiments confirmed that solasonine influences the malignant biological behaviors of osteosarcoma cells. It is speculated that ATP1A1, CLK1, SIGMAR1, PYGM, and HSP90B1 may serve as therapeutic targets for solasonine in the treatment of osteosarcoma. These findings lay the preliminary foundation for elucidating the molecular mechanisms of solasonine’s anti-osteosarcoma effects and suggest that SS could be a potential drug for osteosarcoma treatment.

## 2 Materials and methods

### 2.1 Data collection

The Cancer Genome Atlas (TCGA) database was applied to download the TCGA-OS cohort, which included gene expression profiles, clinical information, and survival information of 85 osteosarcoma (OS) tumor tissue samples (training set 1) (access time: 24-7-2024). When dealing with missing values, we only retain genes expressed in at least 80% of the TCGA-OS data, filter out genes with low expression or excessive missing values, remove samples with a survival time of 0, and remove duplicate samples. Check whether the distribution of survival status is reasonable, remove invalid samples with a survival time of 0, and identify variables that do not conform to the hypothesis through proportional hazards (PH) hypothesis testing. Use the surv_cutpoint function to determine the optimal risk cutoff point to avoid the influence of outliers on grouping. Subsequently, the Gene Expression Omnibus (GEO) database was applied to download the OS-related transcriptome dataset (GSE99671 and GSE39055). GSE99671 (platform: GPL20148, training set 2) consisted of tumour tissue from 18 OS and 18 control bone tissue samples. On the other hand, GSE39055 (platform: GPL14951) was used as the validation cohort, consisting of 37 tumor tissue samples with survival information. GSE99671: Remove genes with expression levels exceeding 50% from all samples and filter out low expression genes to ensure sufficient data to support differential analysis. GSE39055: Use na.omit() to remove incomplete samples when merging expression and survival data, ensuring that expression data samples match clinical data samples exactly.

The MOL2 structure of solasonine was obtained from TCMSP and imported into the PharmMapper database (Z-score > 0) to predict targets for solasonine. In addition, the target names were corrected and unified using the UniProt database, resulting in 93 targets. In the SEA database, 24 targets for solasonine were identified, and in the SwissTargetPrediction database, 103 targets for solasonine were identified. Subsequently, a union of all targets was generated and duplicate genes were removed to obtain 210 targets for solasonine (**Supplementary Table 1**). Subsequently, OS-related disease targets were screened in OMIM, CTD, and DisGeNET databases, resulting in 11, 42852, and 30 targets, respectively. The gene data obtained were combined, and duplicates were removed to obtain the final 28050 OS-related disease targets (**Supplementary Table 2**).

### 2.2 Acquisition of candidate targets

By using the *limma* package (v 1.38.0) (21), we analyzed the differentially expressed genes (DEGs) between OS and control tissue samples in GSE99671 (|log_2_FC|> 0.5, P.adj<0.05). Then the ggplot2 (v 3.4.1) (22) and ComplexHeatmap (v 2.18.0) (23) packages were applied to visualize the results by plotting a volcano plot and heatmap for the top 10 up- and down-regulated genes. Concurrently, the intersection of DEGs, targets corresponding to solasonine, and OS-related disease targets was taken to obtain candidate targets.

### 2.3 Enrichment analysis and construction of protein-protein interaction based on candidate targets

Subsequently, by using clusterProfiler package (v 4.4.4) (24), potential biological functions and pathways on candidate targets were elucidated through Gene Ontology (GO) and Kyoto Encyclopedia of Genes and Genomes (KEGG) (adj.P <0.05). The 10 most significantly enriched pathways in KEGG were selected for display. The 10 most significant items from each category in GO were then selected for display. Additionally, candidate targets were input into the Search Tool for the Retrieval of Interacting Genes/Proteins (STRING) (a confidence score threshold of ≥ 0.4) to explore the protein-level interactions of these genes, which were visualized by using Cytoscape software (v 3.1.1) (25).

### 2.4 Construction and validation of the prognostic model

In TCGA-OS, by using the survival package (v 3.5-3) (26), univariate and multivariate Cox regression analyses (hazard ratio (HR) ≠ 1, *P <0.05*) were performed on candidate targets, results of the regression analyses were separately subjected to proportional hazards (PH) assumption tests (*P > 0.05*). Subsequently, forest plots were drawn using the *forestplot* package (v 2.0.1) (27) to display the results of the univariate and multivariate Cox regression analyses. Finally, the key genes were identified through stepwise regression analysis.

Based on the relative expression levels of key targets and the risk coefficients obtained from stepwise regression analysis, risk scores for OS patients were calculated using the following formula:


$$\text{Risk score}=\sum_{\text{i}=1}^{\text{n}}\text{coef }\left({\text{gene}}_{\text{i}}\right)\times \text{expr }({\text{gene}}_{\text{i}})$$

vThe coefficients (*coef*) were obtained as weights from the stepwise regression analysis. These weights represented the contribution of each gene expression level to the overall risk score, while expression (*expr*) indicated the expression level of the *i*-th gene. Subsequently, OS patients were divided into high-risk and low-risk groups using the optimal cutoff value of the risk score. Next, the survival package (v 3.5-3) was used to draw risk curves and survival status plots to analyze the distribution of OS patients in different datasets. Additionally, the Kaplan-Meier (K-M) curve was plotted to evaluate the overall survival between the 2 groups. The receiver operating characteristic (ROC) curve (1/2/3 years) was visualized to evaluate diagnostic value of the prognostic model (area under the curve (AUC) >0.7) by using the survivalROC package (v 1.0.3) (28). In addition, our risk model was validated in the TCGA-OS cohort.

### 2.5 Nomogram model construction and evaluation

Subsequently, a nomogram was constructed for the key targets in the training set using the rms package (v 6.8-1) (29). The constructed nomogram model was then evaluated through calibration and ROC curves.

### 2.6 Gene set enrichment analysis (GSEA)

In TCGA-OS, differential expression analysis was performed on the 2 groups in the prognostic model using the DESeq2 package (v 3.19) (30), and log_2_FC was calculated and ranked from highest to lowest. Based on the ranking results, using “c2.cp.kegg.v2022.1.Hs.symbols.gmt” as the reference gene set, GSEA analysis was conducted. The top 5 significantly enriched pathways (adj.p <0.05) were then selected for display.

### 2.7 Immune microenvironment analysis

Meanwhile, in TCGA-OS, the ssGSEA algorithm was applied to calculate the differences in immune cell infiltration levels between the high-risk and low-risk groups in each OS patient sample, which were compared to identify differential immune cells (Wilcoxon rank sum test *P <0.05*). Subsequently, differential immune cells in OS were displayed as box plots drawn using the ggplot2 package (v 3.4.1). Additionally, Further investigation of differential immune cells was conducted. Spearman correlation analysis (|cor| > 0.3, *P <0.05*) was conducted on differential immune cells and immune cells, immune cells and key targets, immune cells and risk score. At the same time, The expression differences of 46 immune checkpoints (31) between two groups (*P <0.05*) were then evaluated. Finally, the immune score, stromal score, and ESTIMATE score of each patient sample were calculated using the ESTIMATE algorithm (*P <0.05*).

### 2.8 Structural analysis of key targets and construction of TF-mRNA regulatory network

Subsequently, the gene information and structure of key targets were obtained from the Gene database (https://www.ncbi.nlm.nih.gov/gene/) and the UniProt database. Then, the Exon-Intron Graphic Maker (http://www.wormweb.org/exonintron) was used to input the gene’s 5’UTR, 3’UTR, exons, and introns to obtain a simplified structure of gene transcript. Additionally, the protein domains were illustrated using the IBS tool (http://ibs.biocuckoo.org/) based on target information provided by the UniProt database. Key targets’ transcription factors were identified using FunRich (http://www.funrich.org), and the results were visualized with Cytoscape software (v 3.7.2) (25).

### 2.9 Molecular docking

Molecular docking was performed between solasonine and key targets to determine their binding affinity. The 3D structures of the key target proteins and solasonine were downloaded from the Research Collaboratory for RSCB PDB and PubChem, respectively, and molecular docking was conducted using the CB-Dock database. The binding affinity between solasonine and key targets was determined through molecular docking. RSCB PDB and PubChem were separately applied to download 3D structures of the key target proteins and solasonine, and molecular docking was conducted using the CB-Dock database (docking score < -5 kcal/mol).

### 2.10 Experimental validation of key targets

#### 2.10.1 Cell culture

The human osteoblast cell line hFOB 1.19 and osteosarcoma cell lines 143B, U2Os, Saos, and MG63 (Saiba, Shanghai, China) were maintained in the laboratory of Ningxia Medical University. Each cell line was cultured in its specific growth medium and incubated in a humidified incubator at 37°C with 5% CO_2_ to ensure optimal growth conditions.

#### 2.10.2 Reverse transcription quantitative polymerase chain reaction

Total RNA was extracted using the UltraPure RNA Extraction Kit (CW0581M, CWBIO) according to the manufacturer’s instructions. The concentration and purity of the RNA were then measured. cDNA was synthesized from the RNA using a reverse transcription kit following the provided protocol. For RT-qPCR detection, 2×SuperStar Universal SYBR Master Mix was used as the fluorescent dye. The primers for ATP1A1, CLK1, SIGMAR1, PYGM, HSP90B1, and the internal reference gene β-actin were synthesized by General Biosystems (Anhui, China) Co., Ltd. (**Supplementary Table 3**).

### 2.11 Cell proliferation and viability assays

Reagent and source: Solasonine was purchased from MedChemExpress (MCE, Cat. No.: HY-N0070), CCK-8 detection kit (KeyGEN BioTECH,China,Cat.No.: KGA9305-500). The proliferation and cell viability of 143B cells were detected by CCK 8: until the cell density was grown to about 90%, the cells were passaged, collected, counted, and spread in about 10,000 cells per well in 100μL per well, and cultured in an incubator overnight. The next day, different concentrations of Solasonine were added so that the final concentrations in the wells were 40,20,10,5,2.5,1,0 uM and placed in an incubator for 24 h, 48 h. Then 10μL CCK8 of reagent was added to each well and incubated in an incubator for 2 h. The absorbance of each well was detected by an enzyme marker at 450 nm.

### 2.12 Assessment of cell migration and invasive capacity

#### 2.12.1 Wound healing assays

143 B and MG63 cells were inoculated in 6-well plates overnight, and when the cell density reached 80% fusion, the cells were vertically scratched with a 200μL sterile pipette tip and washed with PBS to remove detached cells. The cells were then incubated with 10μM solasonine for 24h, 48h and cell migration was detected.

#### 2.12.2 Transwell assays

The matrix gel (KeyGEN BioTECH, Jiangsu, China) was prediluted at 1:8 in the Transwell upper chamber (PC membrane 6.5 mm, pore diameter 8 microns) and polymerized at 37°C for 4 h. When 143B cells were starved for 24 hours, 100μL of cell suspension was added to the upper chamber (serum-free medium). After the cells were attached, 200μL of serum-free medium containing 10μM of solasoline were added, and the lower chamber was filled with a complete medium containing 20% FBS. After 24 h of incubation, cells were fixed with 4% paraformaldehyde and stained with 0.1% crystal violet, and five randomly were selected.

### 2.13 Statistical analysis

The R programming language (v 4.2.2) was used for bioinformatics analyses. Differences between two groups were compared by the Wilcoxon rank sum test (*P* <0.05). The log-rank test was used to evaluate the differences between groups in survival analysis (*P* <0.05). All experiments were repeated in triplicates. Data were presented as mean ± SD. Statistical differences between each group were compared using the Student’s t-test. *P* <0.05 was considered statistically significant. The one-way analysis of variance (ANOVA) was used to compare multiple groups. Statistical significance was defined as *P* <0.05.

## 3 Results

### 3.1 Acquisition of 37 candidate targets

**Figure 1:**
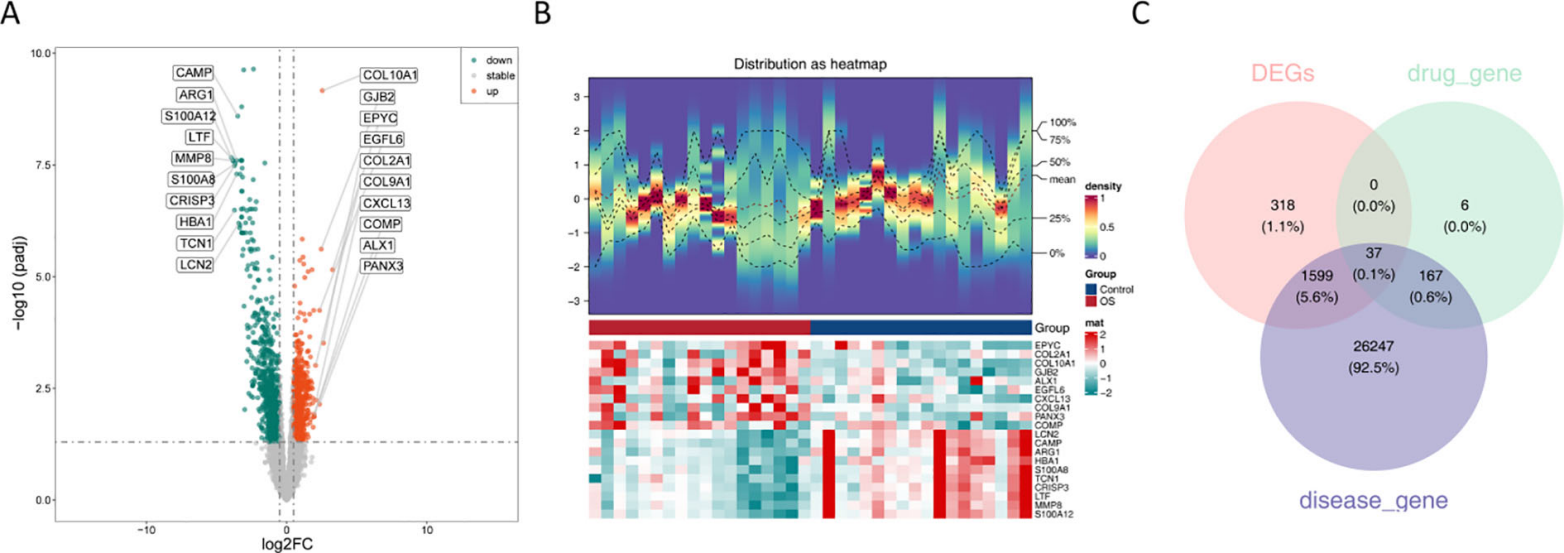
Screening of candidate targets. **(A)** Volcano plots of 1954 DEGs between the OS and control groups; **(B)** Heatmap of expression of 1954 DEGs between OS and control groups; **(C)** The Venn diagram showing 37 targets corresponding to osteosarcoma.

Differential expression analysis was performed using the GSE99671 dataset, including 965 upregulated and 989 downregulated genes in the OS group (**Figure 1A**). Additionally, a heatmap constructed with the top genes demonstrated that these genes could effectively distinguish between the OS and control groups (**Figure 1B**). Finally, the intersection of DEGs, 210 targets corresponding to solasonine, and 28050 OS-related disease targets resulted in 37 candidate targets (**Figure 1C**, **Supplementary Table 4**).

### 3.2 Function enrichment and PPI analysis of candidate targets

**Figure 2:**
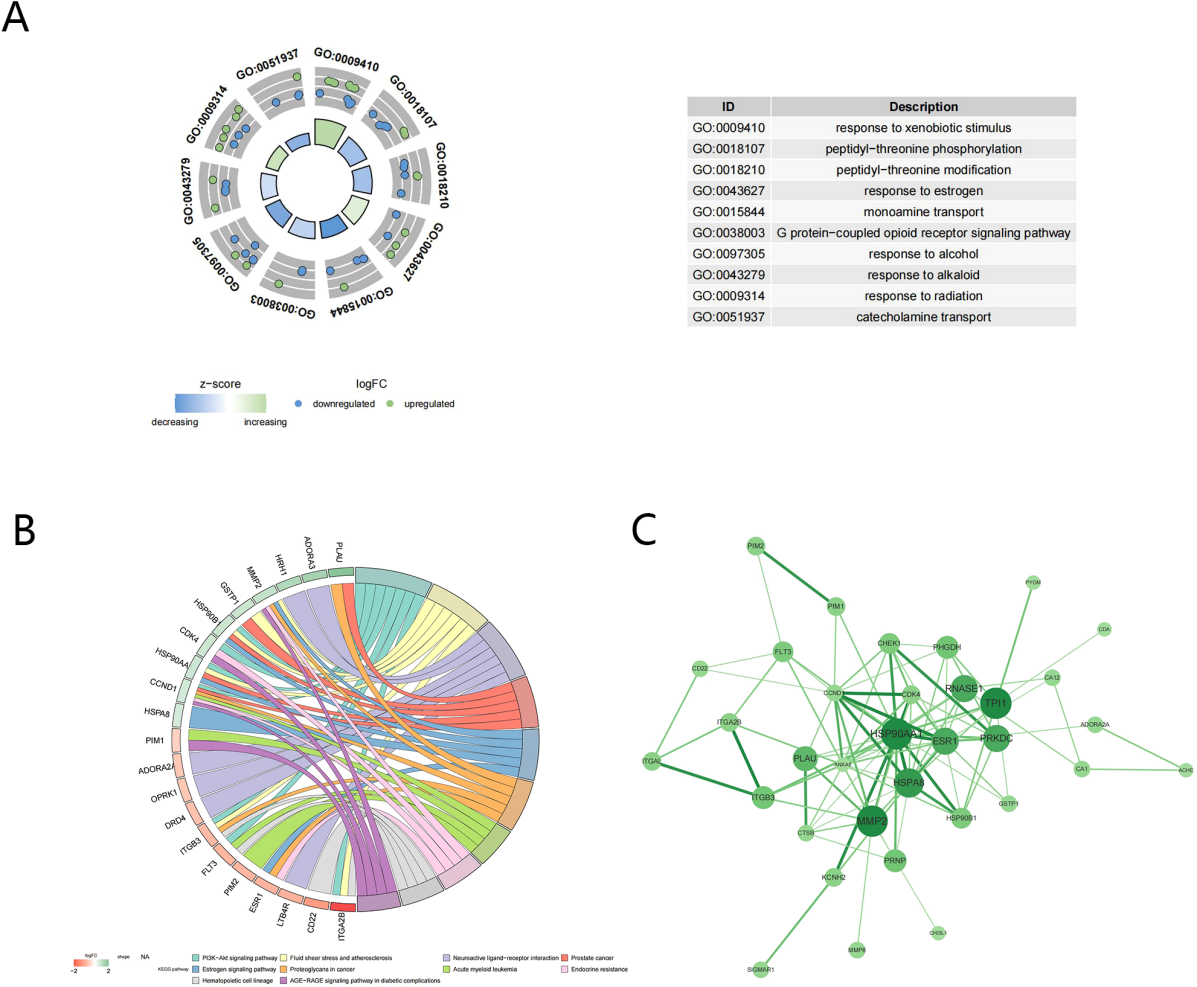
Function enrichment and PPI analysis of candidate targets. **(A)** GO enrichment analysis; **(B)** KEGG enrichment analysis; **(C)** Protein-protein interaction of candidate targets.

The GO analysis conducted on 37 candidate targets separately enriched 95, 29, and 44 terms of biological process (BP), molecular function (MF), and cellular component (CC), such as response to xenobiotic stimulus and peptidyl-threonine phosphorylation (adj.p <0.05) (**Figure 2A**). Additionally, 33 KEGG pathways were enriched, such as th PI3K-Akt signaling pathway (**Figure 2B**). The PPI network consisted of 34 nodes and 96 edges, including HSP90A1, MMP2, HSPA8, and TPI1 (**Figure 2C**).

### 3.3 Identification of key targets and construction of prognostic model

**Figure 3:**
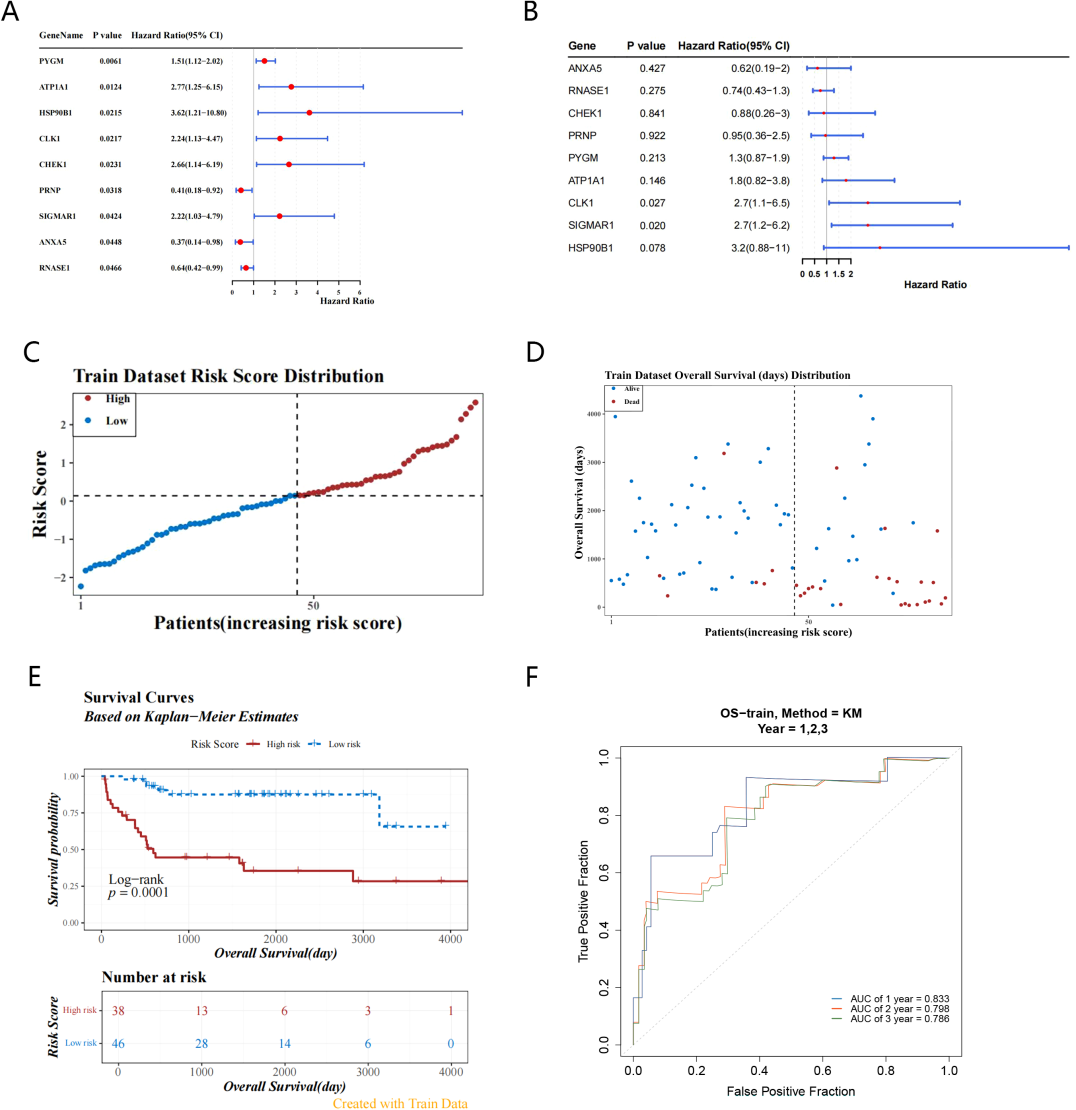
Identification of key targets and construction of prognostic model. **(A, B)** Forest plots demonstrate the acquisition of 9 candidate genes by univariate COX regression analysis and multivariate COX regression analysis based on 37 candidate genes; **(C)** Train dataset risk score distribution based on 5-targets risk scoring model(high-risk: 38 samples, low-risk: 46 samples); **(D)** The survival status distribution map of the high and low risk groups shows that the higher the risk score, the shorter the survival time; **(E)** The training set K-M curve indicate low survival in the high-risk group; **(F)** The ROC curve in the training set (AUC greater than 0.7) suggests that the prognostic model has good predictive performance.

9 survival-related genes were identified after univariate and multivariate Cox regression analyses, and PH assumption tests (*P > 0.05*) were conducted separately for the regression analysis results (**Figures 3A, B**) (**Tables 1**, **2**). Then, through stepwise regression analysis, 5 key targets (ATP1A1, CLK1, SIGMAR1, PYGM, HSP90B1) were finally determined (**Table 3**).

**Table 1:** Univariate pH test results.

id	p
ATP1A1	0.927415916
CLK1	0.651164851
ANXA5	0.126637277
SIGMAR1	0.997159862
PYGM	0.298314958
CHEK1	0.482730991
HSP90B1	0.138982571
RNASE1	0.696916477
PRNP	0.501966222

**Table 2:** Multivariate pH test results.

id	chisq	df	p
ATP1A1	0.345049318	1	0.556929059
CLK1	0.106957524	1	0.7436349
ANXA5	0.34721792	1	0.555692239
SIGMAR1	0.090021544	1	0.764149769
PYGM	0.202826396	1	0.652449068
CHEK1	0.028542881	1	0.865838814
HSP90B1	1.766064941	1	0.183869824
RNASE1	0.701225796	1	0.402372115
PRNP	0.013312638	1	0.908143608

**Table 3:** Risk coefficients of key targets.

id	coef	exp(coef)	se(coef)	z
ATP1A1	0.821685013	2.274328884	0.340209492	2.415232475
CLK1	1.060058641	2.886540254	0.379213922	2.795410665
SIGMAR1	1.104832742	3.018719523	0.430319273	2.567472137
PYGM	0.319977557	1.377096858	0.16187365	1.976711817
HSP90B1	1.024809469	2.786564482	0.56253301	1.821776591

Based on the relative expression levels of key targets and the risk coefficients obtained from stepwise regression analysis, the risk model was constructed: Risk score = 0.8217ATP1A1 + 1.0601CLK1 + 1.1048 SIGMAR1 + 0.3120PYGM + 1.0248HSP90B1. Using the optimal cutoff value (cutpoint = 0.1395293) for the risk score, the 2 groups (high-risk: 38 samples, low-risk: 46 samples) among OS patients were determined (**Figure 3C**). Moreover, the survival status plot indicated that the higher the risk score, the greater the number of deceased OS cases (**Figure 3D**). Concurrently, it was found that, in the high-risk group, patients had lower survival rates through the K-M curve (p = 0.0001) (**Figure 3E**). The ROC curve indicated that the constructed prognostic model could effectively predict the survival rates of OS patients (the AUCs were all greater than 0.7) (**Figure 3F**).

**Figure 4:**
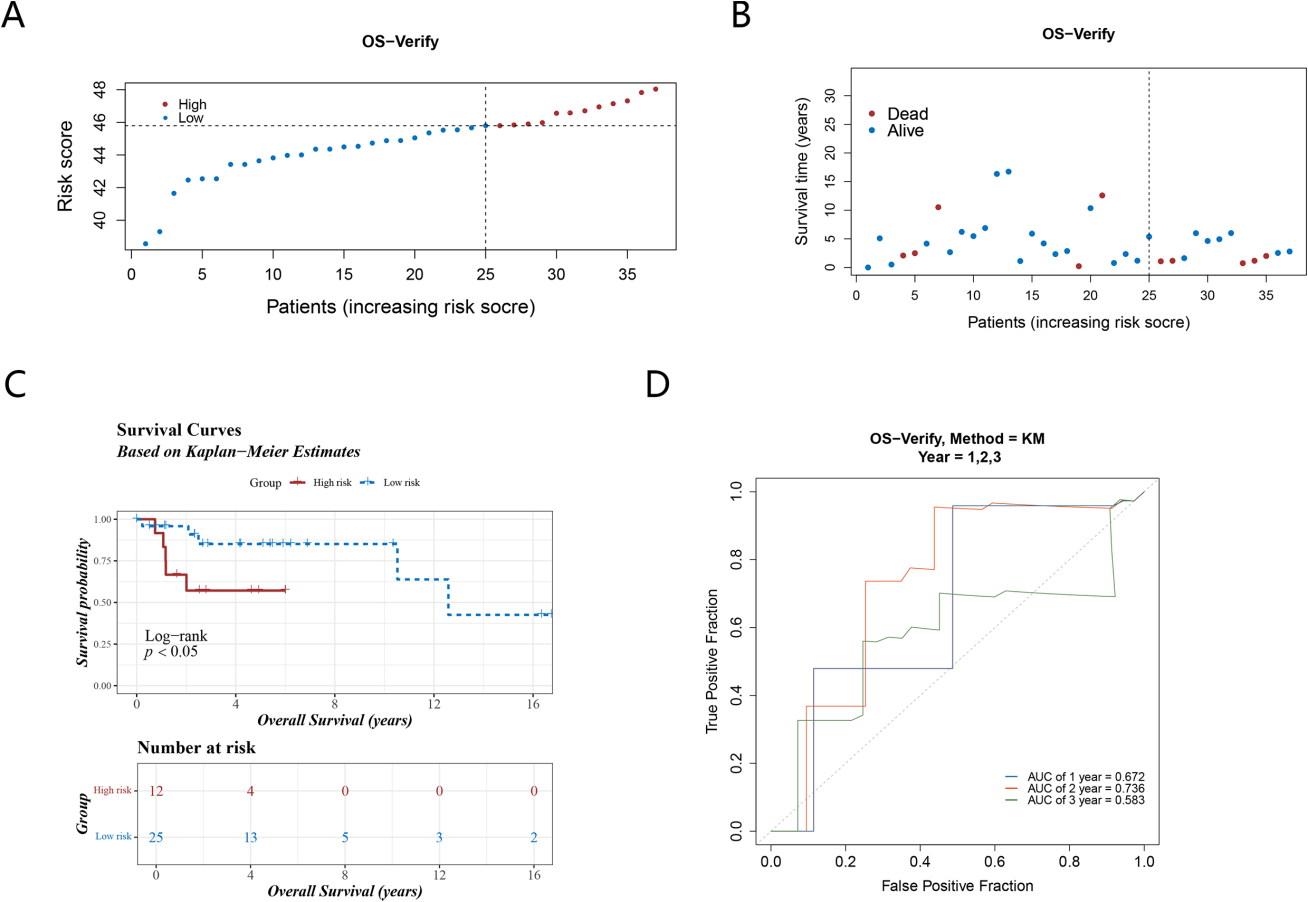
The validation set verifies the reliability of the prognostic model. **(A)** Risk score distribution of the high and low risk groups; **(B)** The survival status distribution of validation set; **(C, D)** The validation set K-M curves and ROC curves illustrate the robust performance of the prognostic model.

Through GSE39055, the reliability of the prognostic model was then validated. A model was applied to calculate optimal threshold (45.79048), 2 groups (high-risk: 12 samples, low-risk: 25 samples) were determined (**Figure 4A**) The results of the survival status plot and the K-M curve (*P <0.05*) were consistent with those of the TCGA-OS (**Figures 4B, C**). Furthermore, the ROC analysis demonstrated AUCs exceeding 0.6 for 1 and 2 years, respectively, while the AUC for 3 years approached 0.6 (**Figure 4D**). These outcomes confirmed the robustness of the risk model in assessing the prognostic risk of OS patients.

### 3.4 Construction and evaluation of OS prediction model

**Figure 5:**
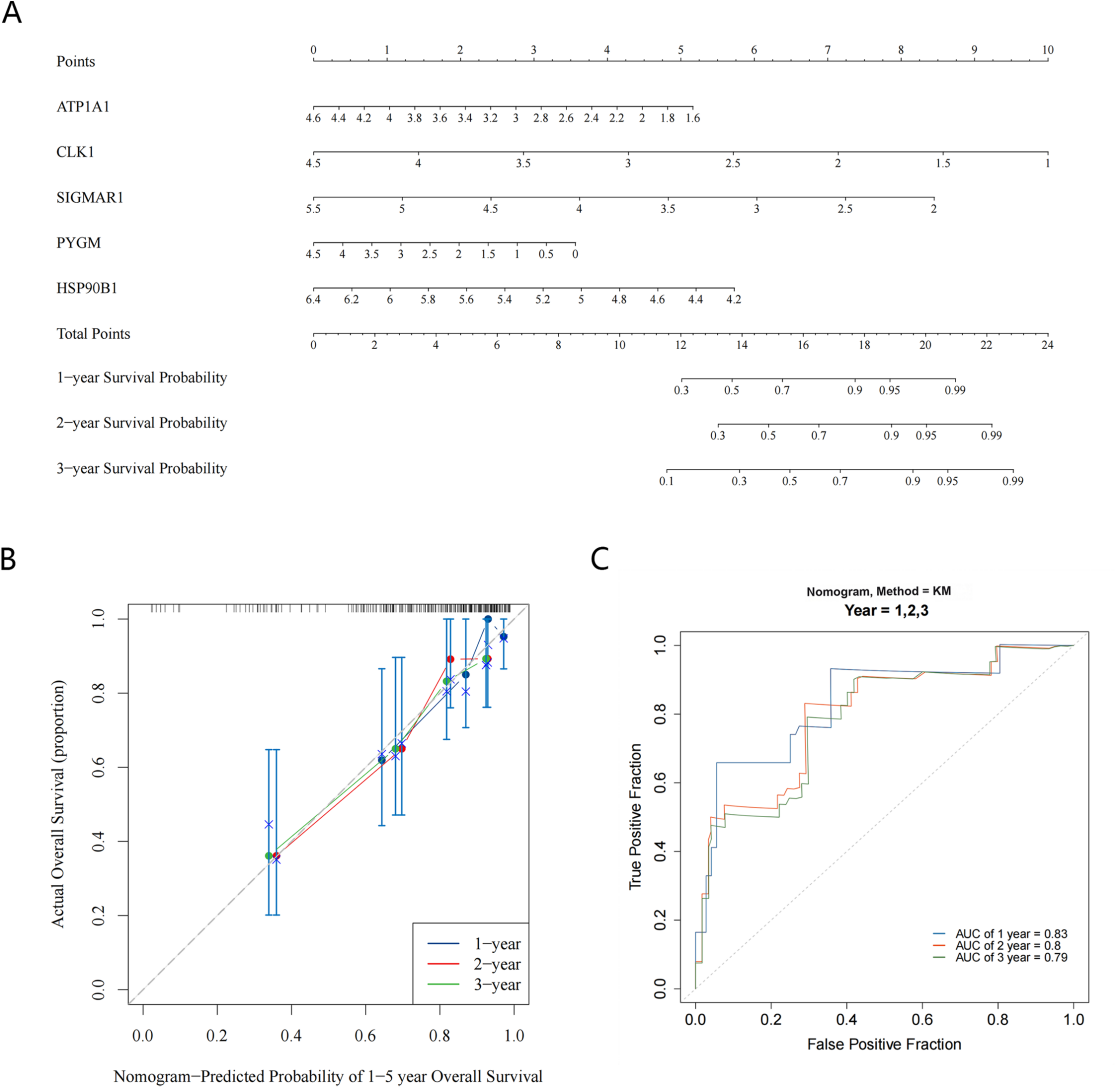
Construction and evaluation of nomogram. **(A)** The Nomogram model shows the value of five key targets in contributing to the prediction of patient survival; **(B)** The calibration curve for the Nomogram predicts patient survival at 1-year, 2-year, and 3-year; **(C)** ROC curves suggest good predictive performance of the nomogram (AUC > 0.7).

Meanwhile, the results of the Nomogram showed that CLK1 had the greatest contribution to patient survival, followed by SIGMAR1, HSP90B1, ATP1A1, and PYGM (**Figure 5A**). The calibration curve showed that the slopes of the nomogram-predicted survival probabilities for different years were close to 1 (**Figure 5B**). Furthermore, the AUC values were all greater than 0.7 (1-, 2-, and 3-year) (**Figure 5C**), indicating that the nomogram had good predictive performance.

### 3.5 Risk score-based GSEA analysis

**Figure 6:**
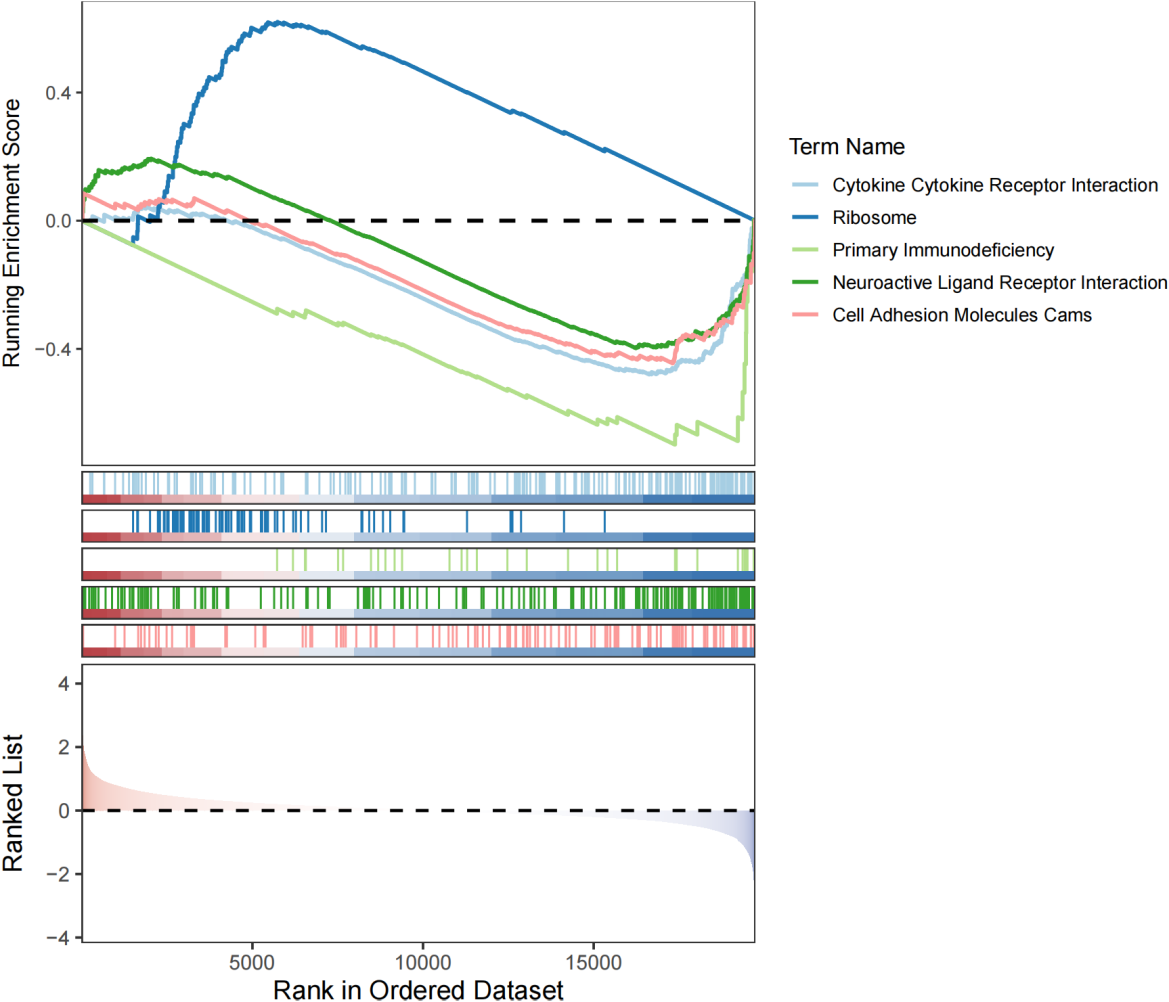
GSEA analysis of the high and low risk groups.

Subsequent GSEA analysis identified 34 pathways that showed significant differences between the 2 groups (adj.p <0.05), such as ribosome (**Figure 6**). These functions still require further investigation.

### 3.6 Description of the immune microenvironment in OS

**Figure 7:**
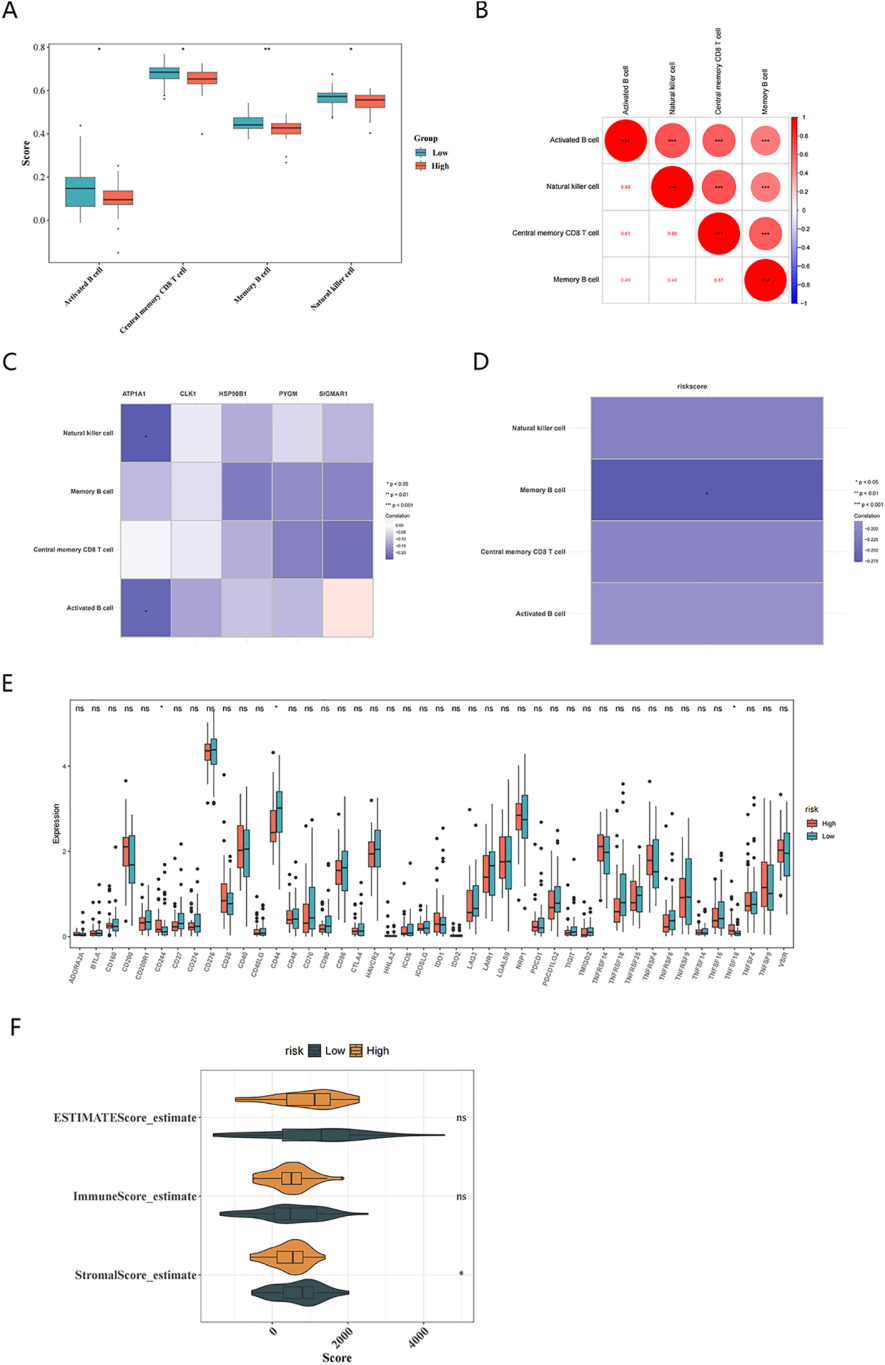
Description of the immune microenvironment in OS. **(A)** Identification of differential immune cells in high and low-risk groups based on risk scores; **(B)** Correlation between differential immune cells; **(C)** Heatmap of correlations between differential immune cells and key targets; **(D)** Heatmap of correlation between risk scores and differential immune cells; **(E)** Immune checkpoint expression between high and low risk groups; **(F)** Immune score, Stromal score (P<0.05), and ESTIMATE score between High and Low Risk Groups.

Subsequently, the Wilcoxon test indicated that activated B cells, memory B cells, natural killer cells, and central memory CD8 T cells in the high-risk group were all significantly less abundant than those in the low-risk group (*P* <0.05) (**Figure 7A**). The correlation analysis between key targets and different immune cells showed that ATP1A1 was weakly negatively correlated with natural killer cells and activated B cells (|cor| < 0.3, *P* <0.05) (**Figure 7B**). ATP1A1 was then weakly negatively correlated with activated B cells and natural killer cells (|cor| < 0.3, *P* <0.05) (**Figure 7C**). The correlation analysis between the risk scores and different immune cells showed that only memory B cells were weakly negatively related to risk scores (|cor| < 0.3, *P* <0.05) (**Figure 7D**). Simultaneously, in the high-risk group the immune checkpoints CD244 and TNFSF18 were significantly overexpressed, while CD44 was significantly underexpressed (**Figure 7E**). Additionally, stromal score of the high-risk group was significantly lower (**Figure 7F**).

### 3.7 Exon-intron and protein structure presentation of key targets

**Figure 8:**
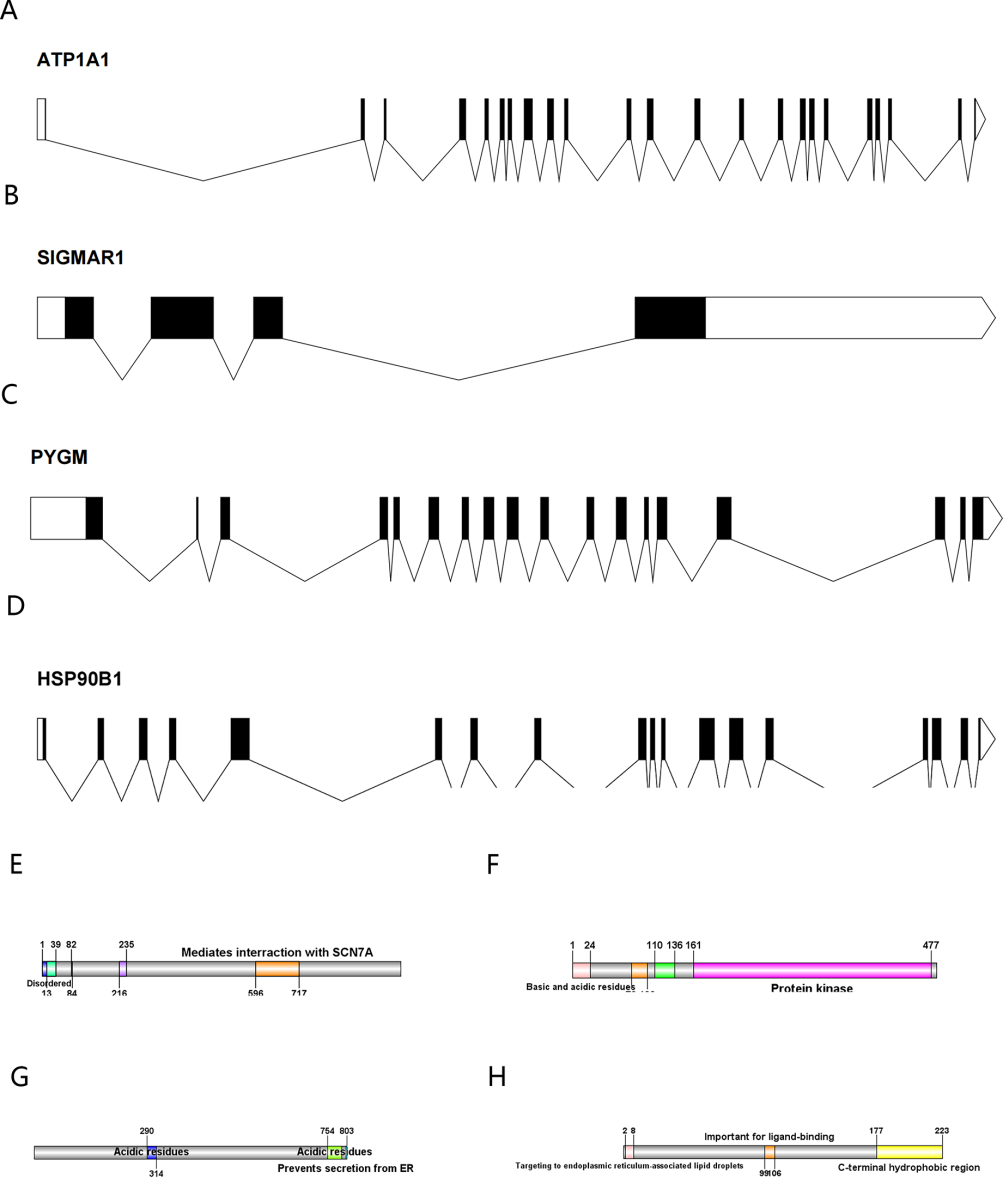
**(A–D)** The exon-intron structures of ATP1A1, SIGMAR1, PYGM, and HSP90B1 (exons are indicated by black rectangles, 5′UTR and 3′UTR by white rectangles, and introns by lines); **(E–H)** The protein structures of ATP1A1, CLK1,HSP90B1, and SIGMAR1 (Color represents structural domains).

The full-length gene transcripts showed that ATP1A1 (**Figure 8A**), CLK1, SIGMAR1 (**Figure 8B**), PYGM (**Figure 8C**), and HSP90B1 (**Figure 8D**) contained 21, 14, 4, 18, and 18 exons, respectively. The full-length gene transcript of CLK1 included non-coding regions, making it impossible to visualize the gene structure of CLK1. The protein structures showed that all proteins contained domains characteristic of themselves (ATP1A1: sodium/potassium-transporting ATPase subunit alpha-1; CLK1: dual specificity protein kinase CLK1; SIGMAR1: sigma non-opioid intracellular receptor 1; PYGM: glycogen phosphorylase, muscle form; HSP90B1: Endoplasmin) (**Figures 8E–H**). Among them, the PYGM gene had no domain information in the Uniprot database, making it impossible to visualize. The gene and protein information of the key targets were summarized (**Table 4**).

**Table 4:** The gene and protein information of the key targets.

Name/gene ID	Aliases	Exon count	Protein names	Location
ATP1A1	CMT2DD, HOMGSMR2	21	Sodium/potassium-transporting ATPase subunit alpha-1	1p13.1
CLK1	CLK, CLK/STY, STY	14	Dual specificity protein kinase CLK1	2q33.1
SIGMAR1	ALS16, DSMA2, HMNR2, OPRS1, SIG-1R, SR-BP, SR-BP1, SRBP, hSigmaR1, sigma1R	4	Sigma non-opioid intracellular receptor 1	9p13.3
PYGM	GSD5	18	Glycogen phosphorylase, muscle form	11q13.1
HSP90B1	ECGP, GP96, GRP94, HEL-S-125m, HEL35, TRA1	18	Endoplasmin	12q23.3

### 3.8 Construction of TF-mRNA regulatory network and molecular docking

**Figure 9:**
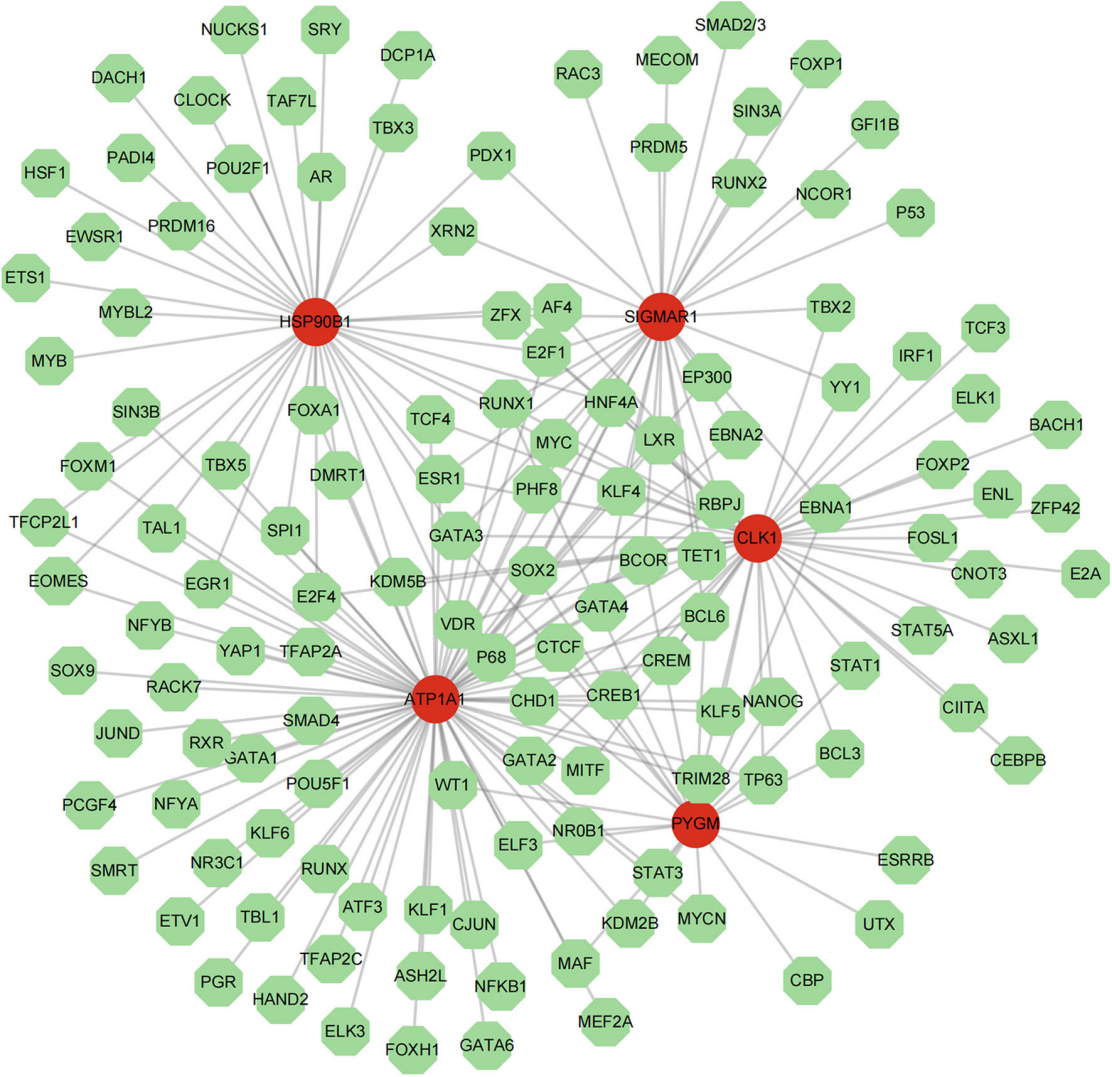
The TF-mRNA regulatory network of 5 key targets.

The TF-mRNA network contained 136 nodes and 214 edges. ATP1A1, CLK1, SIGMAR1, PYGM, and HSP90B1 were predicted to have 78, 42, 33, 22, and 38 transcription factors, respectively. Among them, transcription factors such as GATA3 were found to co-regulate these 5 key targets (**Figure 9**).

**Figure 10:**
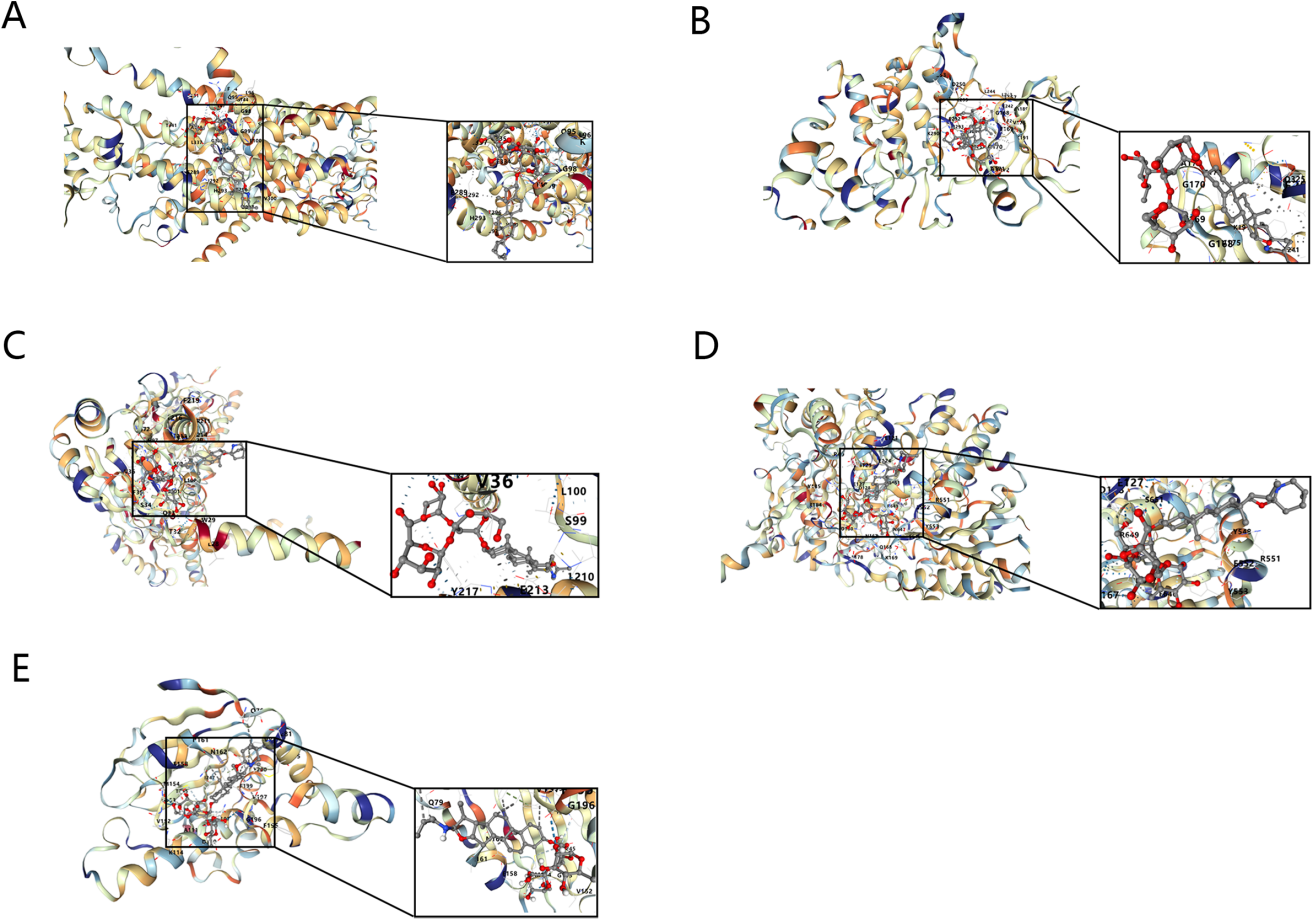
Molecular docking diagrams of Solasonine with ATP1A1 **(A)**, CLK1 **(B)**, SIGMAR1 **(C)**, PYGM **(D)**, and HSP90B1 **(E)**.

It was then found that 5 key targets with solasonine indicated good binding performance (binding energies were all less than -5 kcal/mol) (**Figures 10A–E**) (**Table 5**). Among them, PYGM had the best binding effect with solasonine (docking score = -10.1 kcal/mol).

**Table 5:** Binding energy of key targets with solasodine.

SYMBOL	POB_ID	Docking score(kcal/mol)
ATP1A1	7E1Z	-9.9
CLK1	6Q8K	-8.6
SIGMAR1	5HK2	-9.3
PYGM	1Z8D	-10.1
HSP90B1	4NH9	-7.8

### 3.9 Expression of key targets in osteosarcoma cell lines

**Figure 11:**
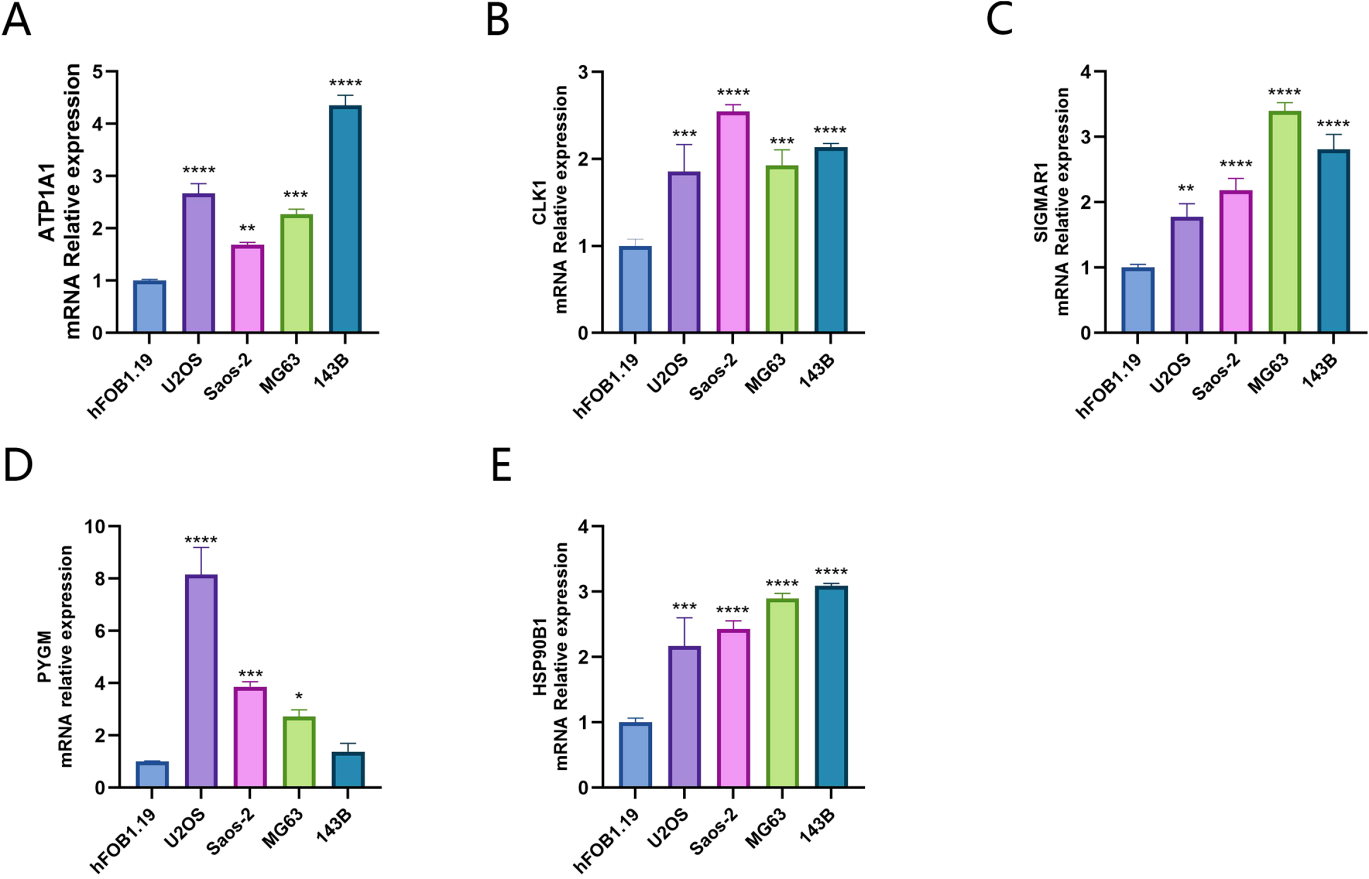
Expression of key targets in osteosarcoma cell lines **(A)** ATP1A1; **(B)** CLK1; **(C)** SIGMAR1; **(D)** PYGM; **(E)** HSP90B1). *p < 0.05, **p < 0.01, ***P< 0.001, ****P < 0.0001.

In the results of the RT-qPCR experiment, compared with the hFOB1.19 cells in the control group, PYGM showed lower expression in the 143B cell line and higher expression in the U2OS, Sao-2 and MG63 cell lines(*P*<0.05). The rest of the target genes all exhibited high expression in the four osteosarcoma cell lines (*P<0.05)*, indicating statistically significant differences(**Figures 11A–E**).

### 3.10 Solasonine affects the malignant biological behavior of osteosarcoma cells

#### 3.10.1 Solasonine inhibits the proliferation of 143B and MG63 cells

**Figure 12:**
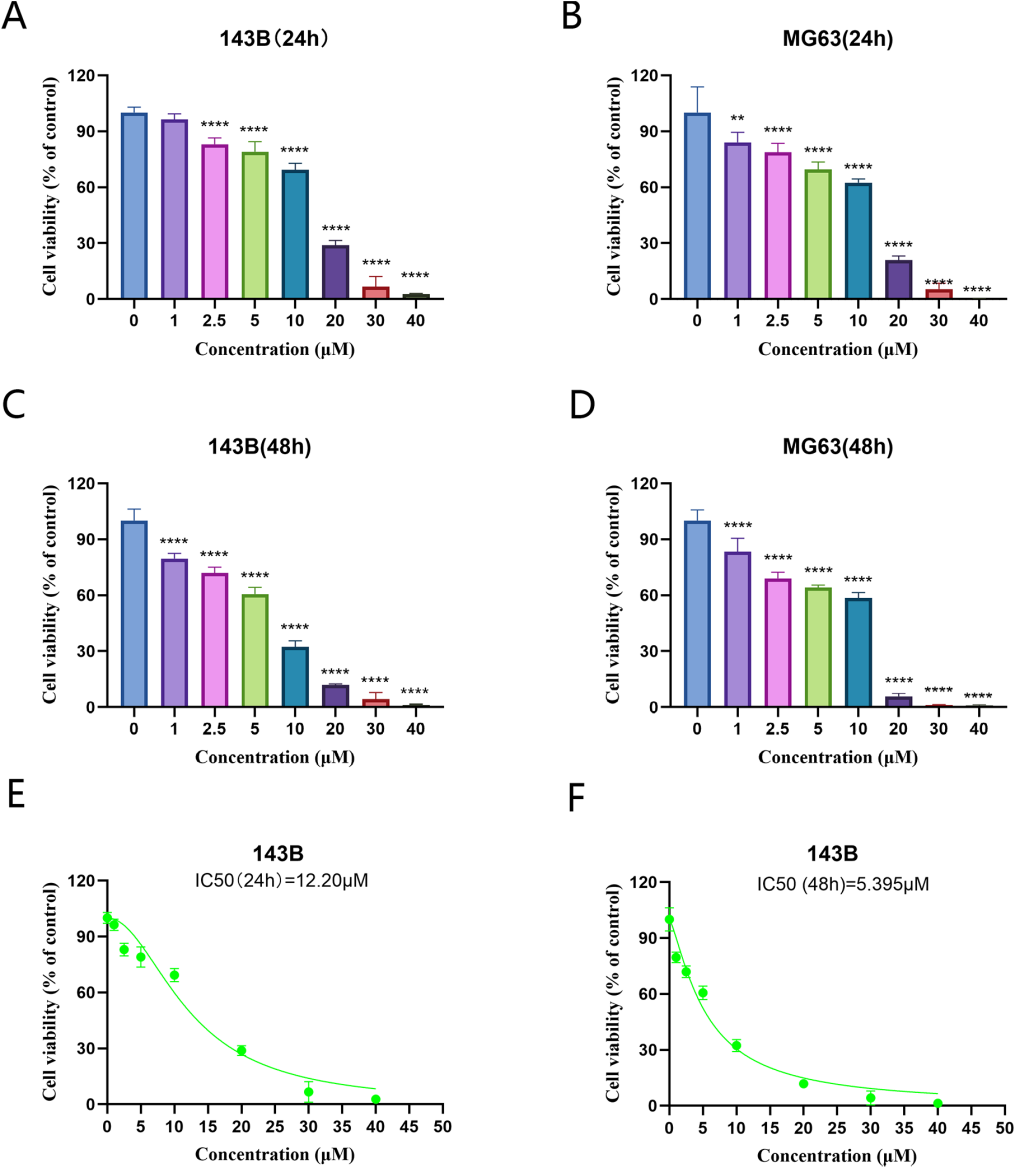
Solasonine inhibited the cell viability in OS cells.143 B **(A, C)**, and MG63 **(B, D)** cells were treated with serial concentrations of SS, and the effects of solasonine on cell viability were measured by cell counting Kit-8 assay at 24 and 48h. **P < 0.01; ****P < 0.0001, vs control. **(E, F)** Viability change curves of 143B cells treated with different concentrations of solasonine.

The results of the CCK 8 assay showed that solasonine inhibited the proliferation of 143B and MG63 cells.Compared with the control group, cell viability decreased significantly in a dose- and time-dependent manner.After 24 h,the difference in 143B cell viability from the concentration of 2.5 μmol/L (*P <*0.05) and gradually decreased with increasing concentration. After 48 h, tthe difference in 143B cell viability from the concentration of 1μmol/L (*P <*0.05) and gradually decreased with increasing concentration (**Figures 12A–D**). The IC50 of 143B cells for 24h and 48h was calculated to be 12.27μmol/L and 5.395μmol/L, respectively (**Figures 12E, F**). The subsequent trials were set with IC50 as reference.

#### 3.10.2 Solasonine inhibited the invasion and migration of 143B and MG63 cells

**Figure 13:**
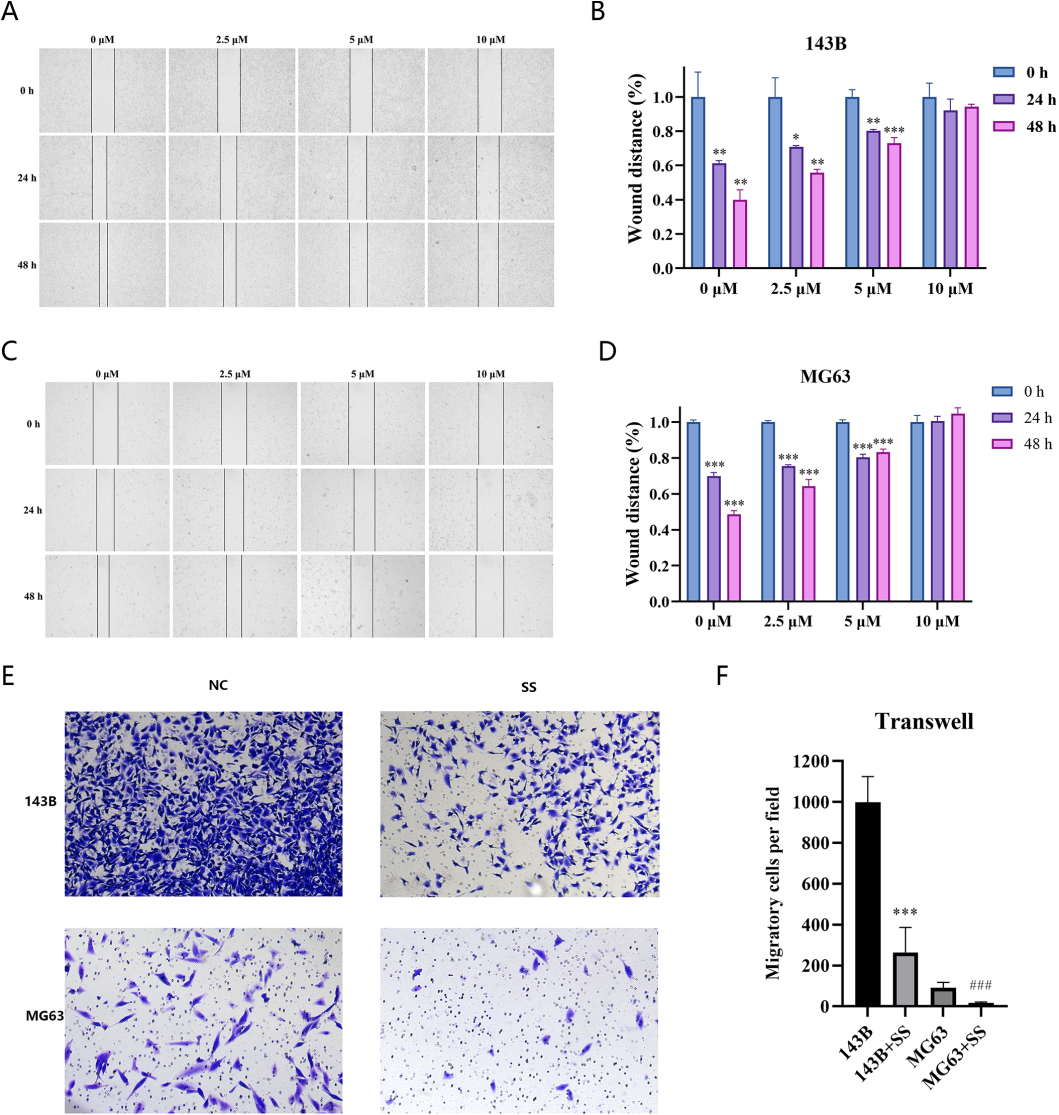
**(A–D)** wound-healing migration of SS-treated 143B **(A, C)**, MG63 **(B, D)** cells and the quantitative assays were examined. **(E, F)** transwell invasion of the SS-treated cell lines and the quantitative assays were examined (***P<0.0001, ###*P*<0.0001).

The inhibitory effects of Solasonine on invasion and migration in 143B and MG63 cells were also examined. The data of wound healing revealed the bigger scratch areas in treated cells (**Figures 13A–D**), indicating the inhibitory effect of Solasonine on migration. The further transwell assay also showed less invaded cells in solasonine-treated groups (**Figures 13E, F**), which was consistent with the wound healing results.

## 4 Discussion

Osteosarcoma is a common primary bone malignancy in children and adolescents (32). In recent years, despite the remarkable development of early diagnosis and treatment of osteosarcoma, the overall survival rate of osteosarcoma patients has not been significantly improved due to its low sensitivity and resistance to chemotherapeutic drugs (33). How to further enhance the sensitivity of osteosarcoma chemotherapy drugs and reduce the occurrence of drug resistance without increasing chemotherapy toxicity and economic burden on patients is still a difficult problem in current clinical treatment. Solasonine is a natural alkaloid, one of the main components of traditional Chinese medicine, Solanum nigrum L.(Long kui). It has various pharmacological effects such as anti-tumor, anti-inflammatory, and neuroprotection. Although previous research (17) has explored Solasonine’s role in osteosarcoma, there is still a lack of comprehensive understanding of all its anti - osteosarcoma targets and the associated molecular mechanisms. In this study, for the first time, we screened the candidate genes of SS against osteosarcoma by network pharmacology combined with transcriptomics and performed KEGG and GO analysis on the candidate genes, as well as PPI network construction to identify five key targets, and the prognostic value of the key targets could be clearly defined by constructing a prognostic model of the key target. The importance of these key targets was demonstrated in the subsequent relevant analyses. Then we further verified the expression of the targets in osteosarcoma cells and the malignant biological behaviors of osteosarcoma cells affected by Solasonine.

The in-depth exploration of the pathological mechanisms of osteosarcoma and its related signaling pathways, the development and metastasis of osteosarcoma are closely related to several cell signaling pathways, including Wnt/β-catenin, PI3K/Akt, RAF/MEK/ERK and mTOR (34), which is consistent with the results of this study.

According to the results of the GO and KEGG analysis in this study, the biological process was enriched to response to xenobiotic stimulus. The molecular function was enriched to carbon-oxygen lyase activity, which suggested that Solasonine may activate the cellular stress defense mechanism to clear the exogenous toxic substances and regulate energy metabolism of osteosarcoma cells and Apoptosis sensitivity. There are also studies confirming that solasonine regulate osteosarcoma glucose metabolism through the Wnt/β-Catenin/Snail pathway. This also proves the reliability of our results.

Wang et al. (35) showed that estrogen receptorαexpression can be used as a prognostic factor to predict the response to chemotherapy and inhibit the proliferation of tumors. The dual enrichment of response to estrogen and estrogen signaling pathway in this study implies that solasonine may intervene with candidate genes to inhibit the proliferation or drug resistance of osteosarcoma through the non-genomic effects mediated by estrogen receptor (ESR1/ESR2) or membrane-associated estrogen receptor (GPER1), which also provides some theoretical bases for the direction of the subsequent research on Solasonine to enhance the sensitivity of chemotherapy.

In addition, the association of serine/threonine kinase (Ser/Thr kinase) with the PI3K-Akt pathway suggests that Solasonine may form an estrogen-kinase-PI3K regulatory axis in inhibiting the progression of osteosarcoma cells by inhibiting the phosphorylation modifications (for example, AKT1 Thr308/Ser473) and the downstream mRNA of AKT1 Thr308/Ser473 and downstream mTOR, thereby inhibiting tumor invasion, a mechanism confirmed in other tumor studies (36).

In previous studies, HSP90AA1 was able to mediate autophagy in osteosarcoma to promote drug resistance (37). Combined with the PPI network in this study, it is hypothesized that Solasonine may synergistically regulate the “stress-responsive-molecular chaperone cluster”(HSP90AA1, HSPA8, HSP90B1) and the”metabolism-invasive synergistic network”(TPI1, MMP2) (38, 39), which could promote apoptosis and inhibit proliferation of osteosarcoma cells, and enhance the sensitivity of chemotherapy.

In this study, five possible key targets of SS against osteosarcoma were finally identified: ATP1A1, CLK1, SIGMAR1, PYGM, and HSP90B1. ATP1A1 belongs to the subfamily of Na+/K+ -ATP enzyme. It has been shown that disruption of ion gradients in tumor cells caused by ATP1A1, can synergize with MAPK pathway inhibitors to promote tumor regression (40, 41). Some studies suggest that ATP1A1 may be used as a diagnostic marker for renal cancer and breast cancer, which is related to the prognosis of tumor (42, 43). CLK1 encodes a member of the CDC2-like (or LAMMER) family of dual-specificity protein kinases, which has been found to influence almost all the aspects of tumor biology including: angiogenesis, apoptosis, cell cycle control, invasion, metastasis, and metabolism (44, 45). SIGMAR1 is a 25kDa stress-activated molecular chaperone protein involved in the regulation of calcium homeostasis, endoplasmic reticulum stress response, mitochondrial function, and autophagy (46). SigmaR1 is overexpressed in cancer samples from colorectal cancer (CRC) patients, is associated with higher tumor grade and promotes tumor invasion and angiogenesis (47, 48).PYGM plays a role in insulin and glucagon signaling as well as insulin resistance pathways involved in the regulation of glycogen levels, and its expression level is closely associated with survival prognosis in many cancers (49, 50). HSP90B1 is a conserved member of the heat shock protein family involved in protein folding and translocation. HSP90B1 is highly expressed in various types of tumors and is usually associated with poor prognosis (51). Inhibition of HSP90B1 expression enhances chemotherapy in breast cancer studies (52). HSP90B1 is a direct target of miR-223 and miR-223 may have a tumor suppressor function in osteosarcoma through the PI3K/Akt/mTOR pathway and could be used in anticancer therapies in osteosarcoma (53). All five targets have unique biological functions, and solasonine may intervene in the development of osteosarcoma cells through these targets. This further demonstrates the effectiveness of the selected target genes, providing effective help for the treatment of OS, and verifying potential therapeutic targets for other malignant tumors.

Based on the above five prediction targets, we constructed a disease prognostic model through TCGA-OS data and verified the generalizability of the prognostic model. In the training and validation sets, the risk model was evaluated by drawing K-M curves and ROC curves, and the results showed that the AUC values of the ROC curves for years 1, 2, and 3 were more than 0.7, which indicated that the constructed model was more effective and that the risk model constructed in this study had good predictive performance. From the results of the Nomogram, CLK1 has the greatest contribution to the prediction of overall survival, followed by SIGMAR1, and the total points derived from the combination of the five targets are more predictive of the prognosis of the disease. This indicates that the Nomogram constructed based on the five targets has a good predictive performance. It can be used to evaluate the prognosis of patients with osteosarcoma, and It also suggests that these five targets are valuable for further research.

The GSEA analysis graph shows the changes in the enrichment scores of different gene sets. Studies have found that the ribosomal protein RPL7A is significantly downregulated in osteosarcoma samples. Low RPL7A expression is associated with elevated serum alkaline phosphatase (ALP) levels in osteosarcoma patients and serves as an independent predictor of poor prognosis in lung metastasis cases (54). Additionally, research has shown that knocking down the ribosomal protein S15A (RPS15A) using a lentiviral-mediated RNA interference system can significantly inhibit the proliferation and colony formation of human osteosarcoma U2OS cells, causing them to arrest in the G0/G1 phase of the cell cycle (55). In this study, ribosomes were significantly enriched in the high-risk group, suggesting that ribosome-related biological processes may be more active in high-risk osteosarcoma, promoting protein synthesis and providing a material basis for rapid tumor cell proliferation, invasion, and metastasis. Moreover, among our predicted targets, such as HSP90AA1, HSP90B1, which are related to the protein synthesis process, so we hypothesize that SS acts on the targets to regulate protein synthesis, which in turn affects the progression of osteosarcoma. Of course, there is no single mechanism for the anti-osteosarcoma effect of SS, and the mechanism of action may be multifaceted, involving multiple biological processes such as protein synthesis (ribosome-related), immune regulation (cytokines, primary immunodeficiency-related), neuromodulation and cell adhesion. There may be interactions and synergistic regulation between these targets and pathways, which together affect the development of osteosarcoma.

In the target-based analysis of the immune microenvironment for high and low -risk subgroups, the tumor microenvironment in the high-risk group was characterized by immunosuppression (decreased immune cells, abnormal immune checkpoints) and alterations in the stromal microenvironment. Abnormal activation of immune checkpoints enables tumor cells to evade recognition and attack by the host immune system and promotes tumor progression (56, 57). Moreover correlation analysis of key targets with different immune cells showed that ATP1A1 was moderately negatively correlated with natural killer cells and activated B cells. These findings provide a theoretical basis for therapeutic strategies targeting the tumor immune microenvironment and the ATP1A1 pathway, but further experiments are needed to validate the function and mechanism of ATP1A1. There were significant differences in the matrix components of the tumor microenvironment between high and low risk groups, suggesting that stromal cells and their microenvironment may be involved in the malignant progression and prognostic differentiation of osteosarcoma. It provides a direction for further study of the mechanism and therapeutic intervention of stromal microenvironment in osteosarcoma.

To explore the molecular binding of Solasonine to candidate genes, we did gene structure analysis and molecular docking of 5 targets. In the TF-mRNA network, GATA3, which regulates 5 key targets, is an oncogenic factor and has been reported to be lowly expressed in osteosarcoma, inhibiting OS progression and metastasis by regulating slug (58, 59). In molecular docking, when the binding energy was less than -5 kcal/mol, it indicated a good binding ability, and the 5 key targets had a better binding performance with SS, among which PYGM and SS had the highest binding energy, which indicated that it had the best binding effect.

The combined analysis of transcriptomics and network pharmacology enables deeper insights into the potential targets of solasonine for the treatment of osteosarcoma. As verified by some experiments, the five targets were highly expressed in osteosarcoma cell lines, suggesting that the five targets may jointly serve as potential diagnostic targets for osteosarcoma, Their importance for the development of osteosarcoma needs to be verified by further experiments. Meanwhile, in the drug-intervention osteosarcoma cell phenotype experiment, solasonine is able to inhibit the malignant biological behavior of osteosarcoma. The experimental results showed that 143B cells had a stronger migration and invasion ability than MG63 cells, and the migration ability was reduced after drug action, indicating the complexity of the migration regulation mechanism between the two.

## 5 Conclusion

Based on network pharmacology and transcriptomics, we identified ATP1A1, CLK1, SIGMAR1, PYGM, and HSP90B1 as the key targets of solasonine that influence the progression of osteosarcoma cells. Solasonine, the main component of Solanum nigrum L. (Long kui), targets multiple pathways to regulate various biological behaviors in osteosarcoma cells, including proliferation, apoptosis, migration, and invasion. SS can be a potential drug for the treatment of osteosarcoma.

## 6 Limitations

Due to resource limitations, the main limitation of this study is that the depth of mechanism verification is insufficient: only RT-qPCR was used to verify the high expression of the target at the mRNA level, but no protein level verification was conducted by Western blot, and no knockdown/overexpression experiments were carried out to clarify the causal relationship between the target and the effect of australoxamine.

## 7 Future directions

Future research can be expanded in three areas: (1) Verify the expression of target proteins using Western Bloting and immunofluorescence, and observe changes in cell phenotypes through shRNA knockdown experiments; (2) Validate the function of the target in multiple osteosarcoma cell lines and nude mouse models, and correlate the expression in clinical samples with prognosis; (3) Enhance the synergistic effect of solasonine with chemotherapeutic drugs to improve chemotherapy sensitivity and reduce toxicity; (4) Design solasonine derivatives based on the target structure, optimize their pharmacokinetic properties, and promote their clinical application. These studies will solidify the value of targets predicted by network pharmacology and provide a more comprehensive evidence base for the anti-osteosarcoma mechanism of solasonine.

## Data Availability

The raw data supporting the conclusions of this article will be made available by the authors, without undue reservation.
