# Correction: Integrating network pharmacology and transcriptomics to identify solasonine’s anti-osteosarcoma targets and experimental validation

**DOI:** 10.3389/fonc.2025.1682157

**Published:** 2025-09-29

**Authors:** Daihao Wei, Minyu Li, Dawei Chu, Jiandang Shi

**Affiliations:** ^1^ Department of Orthopedic, General Hospital of Ningxia Medical University, Yinchuan, Ningxia Hui Autonomous Region, Yinchuan, China; ^2^ The First School of Clinical Medicine, Ningxia Medical University, Yinchuan, Ningxia Hui Autonomous Region, China

**Keywords:** solasonine, osteosarcoma, network pharmacology, transcriptomics, experimental validation

There was a mistake in **Figure 1** as published. We modified the relevant parameters according to the opinions of the review experts and generated a new **Figure 1**. The corrected **Figure 1** appears below.

The original version of this article has been updated.

## Figures and Tables

**Figure 1:**
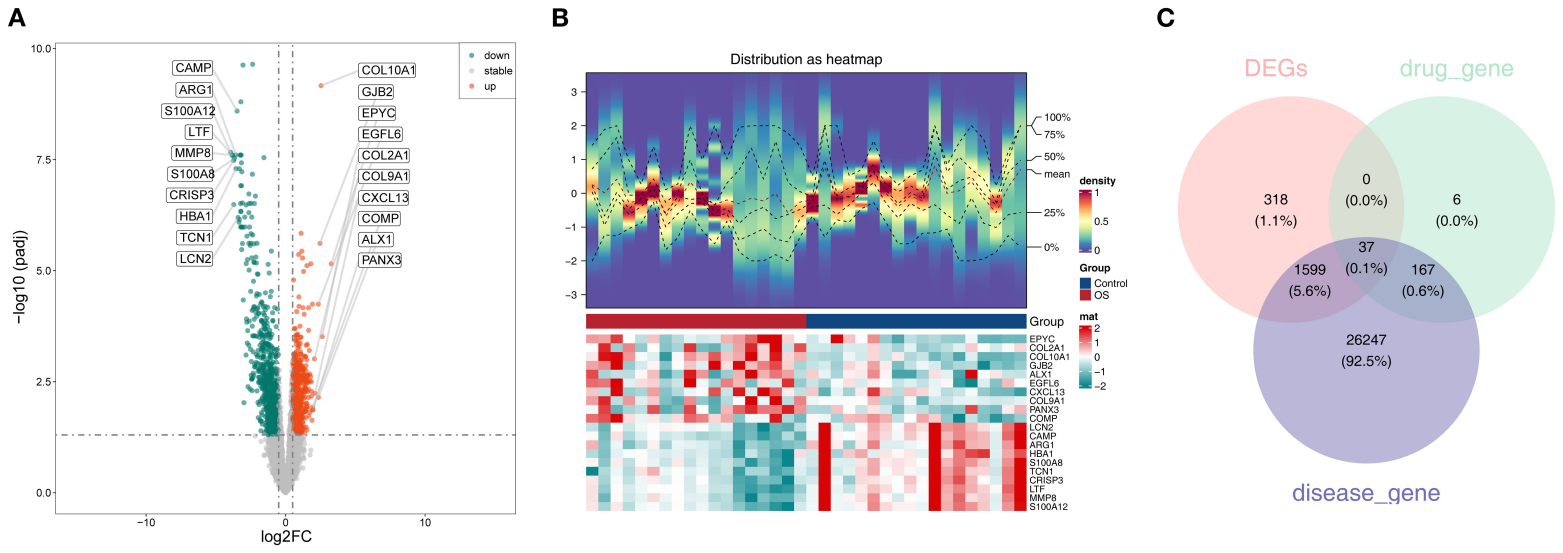
Screening of candidate targets. **(A)** Volcano plots of 1954 DEGs between the OS and control groups; **(B)** Heatmap of expression of 1954 DEGs between OS and control groups; **(C)** The Venn diagram showing 37 targets corresponding to osteosarcoma.

